# A High-Fat Diet Increases Activation of the Glucagon-Like Peptide-1-Producing Neurons in the Nucleus Tractus Solitarii: an Effect that is Partially Reversed by Drugs Normalizing Glycemia

**DOI:** 10.1007/s10571-021-01079-2

**Published:** 2021-04-03

**Authors:** Grazyna Lietzau, Stelia Ntika, Hiranya Pintana, Linda Tracy, Thomas Klein, Thomas Nyström, Vladimer Darsalia, Cesare Patrone, Camilla Krizhanovskii

**Affiliations:** 1 Department of Clinical Science and Education, Karolinska Institutet, Sodersjukhuset Internal Medicine, 118 83 Stockholm, Sweden; 2 Faculty of Medicine, Department of Anatomy and Neurobiology, Medical University of Gdansk, Gdansk, Poland; 3 Department of Molecular Medicine and Surgery, Karolinska Institutet, Stockholm, Sweden; 4 Department of Research, Södertälje Hospital, Södertälje, Sweden; 5 Boehringer Ingelheim Pharma GmbH & Co KG, Biberach, Germany

**Keywords:** Diabetes, Dipeptidyl peptidase-4 inhibitors, GLP-1, Nucleus tractus solitarii, Obesity, Sulfonylurea

## Abstract

Glucagon-like peptide-1 (GLP-1) is a peripheral incretin and centrally active peptide produced in the intestine and nucleus tractus solitarii (NTS), respectively. GLP-1 not only regulates metabolism but also improves cognition and is neuroprotective. While intestinal GLP-1-producing cells have been well characterized, less is known about GLP-1-producing neurons in NTS. We hypothesized that obesity-induced type 2 diabetes (T2D) impairs the function of NTS GLP-1-producing neurons and glycemia normalization counteracts this effect. We used immunohistochemistry/quantitative microscopy to investigate the number, potential atrophy, and activation (cFos-expression based) of NTS GLP-1-producing neurons, in non-diabetic *versus* obese/T2D mice (after 12 months of high-fat diet). NTS neuroinflammation was also assessed. The same parameters were quantified in obese/T2D mice treated from month 9 to 12 with two unrelated anti-hyperglycemic drugs: the dipeptidyl peptidase-4 inhibitor linagliptin and the sulfonylurea glimepiride. We show no effect of T2D on the number and volume but increased activation of NTS GLP-1-producing neurons. This effect was partially normalized by both anti-diabetic treatments, concurrent with decreased neuroinflammation. Increased activation of NTS GLP-1-producing neurons could represent an aberrant metabolic demand in T2D/obesity, attenuated by glycemia normalization. Whether this effect represents a pathophysiological process preceding GLP-1 signaling impairment in the CNS, remains to be investigated.

**Supplementary Information:**

The online version contains supplementary material available at 10.1007/s10571-021-01079-2.

## Introduction

Glucagon-like peptide-1 (GLP-1) is a peptide produced in the intestine and in nucleus tractus solitarii (NTS) in the brainstem (Muller et al. 2019). GLP-1 binds to its receptor (GLP-1R) and its effects on target tissues [i.e., pancreas and central nervous system (CNS)], include improvement of glucose tolerance induction of satiety/inhibition of food intake, gastric emptying (Drucker 2018), neuroprotective actions (Darsalia et al. 2017; Gault and Holscher 2018), and modulatory effects on learning and memory (During et al. 2003).

Peripheral GLP-1 and GLP-1R agonists (GLP-1RA) used in the treatment of type 2 diabetes (T2D) to regulate hyperglycemia, readily diffuses across the blood–brain barrier (BBB) in animal models (Hunter and Holscher 2012), exerting positive effects on insulin secretion and glucose tolerance, at least in part, through CNS GLP-1R activation (Larsen et al. 1997). However, extensive transfer of GLP-1RA across the BBB in rodents is debated and indicated to be minimal in humans with T2D (Christensen et al. 2015). Furthermore, rapid degradation of GLP-1 at the N-terminal end by dipeptidyl peptidase-4 (DPP-4) (Kieffer and Francis Habener 1999) renders it unlikely that significant amounts of active, gut-derived GLP-1 reach the CNS.

Located in NTS, GLP-1-producing neurons that project to various regions of the brain (Llewellyn-Smith et al. 2011; Larsen et al. 1997) expressing the GLP-1R (Graham et al. 2020; Merchenthaler et al. 1999; Cork et al. 2015) are surfacing as important regulators of both peripheral and central GLP-1-mediated effects. Interestingly, in relation to central GLP-1-mediated effect of GLP-1, Holt and colleagues have recently reported that NTS-produced GLP-1 can specifically modulate a satiation/satiety circuit under stress conditions and after large meals (Holt et al. 2019). Consequently, there is an increased need to better understand the role of these cells under physiological and pathophysiological conditions such as T2D.

The aim of this study was to compare the effects of a standard “healthy” diet versus an “unhealthy” obesogenic diet inducing T2D, on the function of GLP-1-producing neurons in NTS. The used obesogenic diet included high-fat content but also differed from the standard diet in terms of composition and amount of energy coming from other energy sources. Specifically, we investigated whether this obesogenic diet inducing T2D could affect the number, induce atrophy, and/or induce changes in neuronal activation of GLP-1-producing neurons. We also investigated whether sustained glycemia normalization could counteract such effects.

## Materials and Methods

### Animal Model and Experimental Design

In accordance with the guidelines aiming to improve the ethical use of animals in experimental research (3R principle) (Balls 2009), the material (brain and plasma) came from the same mice cohort as recently used for another study. Therefore, part of the metabolic data has already been published (Lietzau et al. 2020).

Twenty-five, male C57Bl/6J mice (Charles River, Germany) were housed under controlled conditions, with ad libitum access to food and water. All applicable international, national, and institutional guidelines for the care/use of animals were followed. All experimental procedures were in accordance with the ethical standards of the Karolinska Institutet (Ethical Approval No. S7-13).

Two-month-old mice were randomly assigned to 4 experimental groups. The potential effect of obesity-induced T2D on GLP-1-producing neurons and neuroinflammation in the NTS was determined by comparing outcome parameters in mice fed an obesogenic diet enriched in fat (high-fat diet (HFD): 54% calories from fat, ssniff® E15126-34, Germany) (*n*=6) for 12 months with age-matched mice fed a standard diet (ENVIGO 2018, Italy; SD) (*n*=7). The potential effect of sustained glycemia normalization was determined by adding two groups: HFD-fed mice administered (in food) with either linagliptin (average dose of 5–7 mg/kg b.w. per day; HFD-Lina) (*n*=6) or glimepiride (average dose of 2–4 mg/kg b.w. per day; HFD-Gli) (*n*=6) for 3 months before sacrifice (between month 9 and 12). Both drugs are clinically prescribed for the treatment of T2D, but they have different mechanisms of action. Linagliptin is a dipeptidyl peptidase-4 inhibitor (DPP-4i) that prevents degradation of endogenous GLP-1, which results in increased insulin secretion/sensitivity (Deacon and Holst 2013). Additionally, DPP-4i also have neuroprotective action (reviewed in Darsalia et al. 2019; Chalichem et al. 2017). Sulfonylureas, such as glimepiride, induce direct pancreatic insulin secretion (Khunti et al. 2018). We hypothesized that an effect induced in NTS by both drugs would suggest that it is related with glycemia regulation, but induced only by linagliptin would suggest another, glycemia-independent mechanism.

### Body Weight, Glycemia, DPP-4i Activity, and GLP-1 Levels

Body weight and fasting blood glucose were measured in all mice. Plasma DPP-4 enzyme activity and total active GLP-1 levels were determined (fed state) by EIA and ELISA, respectively (MesoScale Discovery, USA).

### Immunohistochemistry and Quantitative Microscopy

An immunofluorescence staining protocol to quantify GLP-1-producing neurons was applied (Supplementary file) using a NewCast system (Visiopharm, Denmark), connected to Olympus BXS51 microscope (Olympus, Japan). Activation of GLP-1-producing neurons was assessed by quantifying GLP-1/cFos+ cells (Rinaman 1999). To evaluate potential atrophy, mean volume of GLP-1/cFos+ cells was measured using the nucleator technique (Gundersen et al. 1988). Potential activation of neuroinflammation in the NTS was quantified as density/mean volume of Iba-1+ microglia cells and total number of CD68+ microglia cells. Morphological analyses were performed by a blinded experimenter.

### Statistical Analysis

Data were checked by the Shapiro–Wilk normality test. All studied parameters were analyzed as follows:

1. SD *versus* HFD group comparisons were performed using unpaired, two-tailed *t* test with Welch’s correction.
2. HFD *versus* HFD-Lina *versus* HFD-Gli comparisons were performed using ordinary one-way ANOVA followed by uncorrected Fisher’s LSD test.

Data are expressed as mean ± SD. p < 0.05 were considered statistically significant. All data were analyzed by GraphPad Prism 8 (USA).

## Results

**Fig 1:**
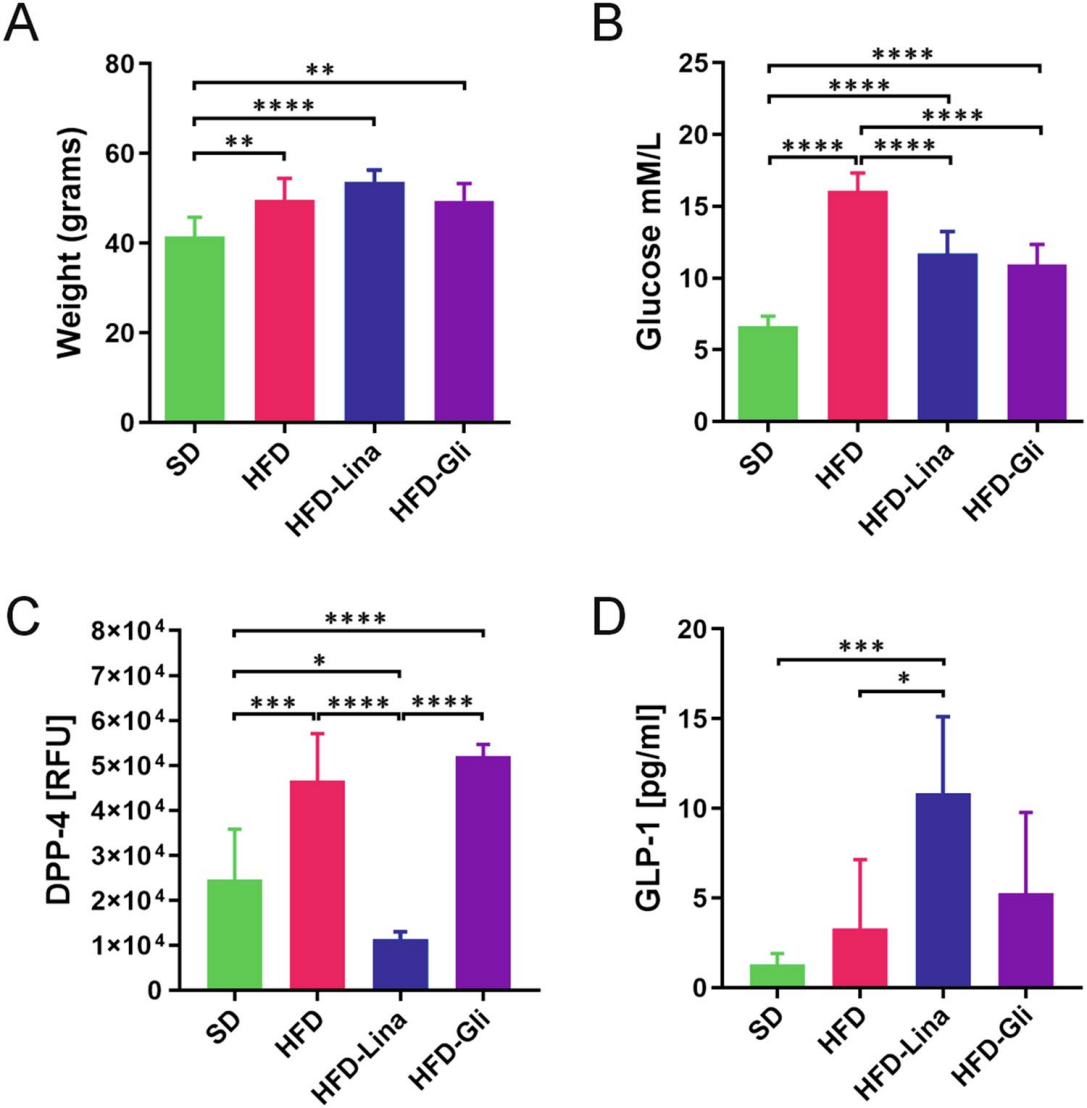
HFD induces obesity and hyperglycemia. Both linagliptin and glimepiride reduce hyperglycemia with no effect on body weight. Body weight (**a**), plasma glucose after 10 hours of fasting (**b**), DPP-4 activity (**c**), and GLP-1 concentration (**d**) in SD and HFD-fed mice, and HFD-fed mice treated either with linagliptin (5–7 mg/kg/b.w. per day) or glimepiride (2–4 mg/kg/b.w. per day) for 3 months. All data are presented as mean ± SD. Unpaired two-tailed *t* test with Welch’s correction was used to compare SD vs. HFD group. Ordinary one-way ANOVA followed by uncorrected Fisher’s LSD test was used to compare HFD vs. HFD-Lina vs. HFD-Gli groups. ¤ denotes *p* < 0.05, ** denotes *p*  <  0.01, ****,¤¤¤¤,&&&& denote *p* < 0.0001, *n*= 4–7. *DPP-4* dipeptidyl peptidase-4 inhibitor, *Gli* glimepiride, *GLP-1* glucagon-like peptide-1, *HFD* high-fat diet, *Lina* linagliptin, *SD* standard diet

Twelve months of HFD intake increased body weight compared to SD-fed mice (*p*=0.0055) (Fig. 1a). Neither 3-month treatment with linagliptin nor glimepiride had any effect on the body weight (Fig. 1a). HFD feeding also resulted in fasting hyperglycemia (*p* < 0.0001 compared to the SD), normalized by both anti-diabetic drugs (*p* < 0.0001 compared to HFD-Lina and HFD-Gli) (Fig. 1b). In linagliptin-treated mice, the plasma activity of DPP-4 enzyme was, as expected, decreased compared to both the HFD and HFD-glimepiride groups (*p* < 0.0001 for both comparisons) (Fig. 1c). Furthermore, linagliptin-treated mice showed a higher concentration of active GLP-1 compared to HFD (*p* < 0.0157) and a similar trend, although not statistically significant (*p*=0.1234), was observed in comparison to the HFD-Gli group (Fig. 1d). Neither difference in DPP-4 enzymatic activity nor GLP-1 plasma concentration was detected between the HFD and HFD-Gli mice (*p*=0.2431 and *p*=0.5973, respectively) (Fig. 1c, d).

**Fig 2:**
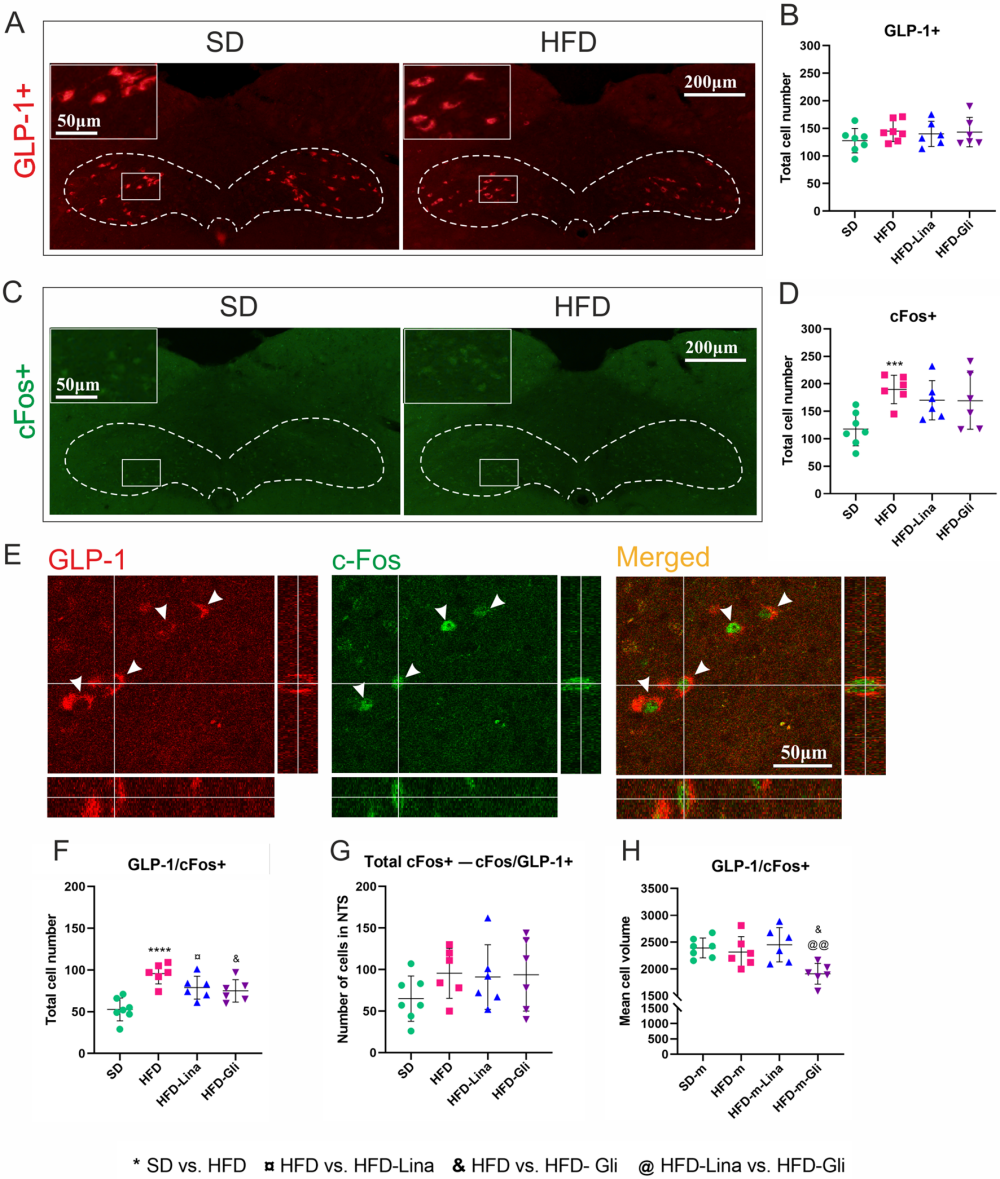
HFD induces activation of GLP-1-producing cells in the NTS. This effect is partially normalized by linagliptin and glimepiride. Representative microphotographs (**a**), and number of GLP-1-producing neurons (**b**) in NTS of SD and HFD-fed mice, and HFD-fed mice treated with linagliptin or glimepiride for 3 months. Representative microphotographs (**c**), and number of cFos+ cells (**d**) in NTS, in the four experimental groups. Rectangles indicate zoomed areas. Representative confocal images with orthogonal reconstruction of GLP-1/cFos+ neurons (arrowheads indicate double-positive cells) (**e**), and number of GLP-1/cFos+ cells in NTS (**f**). Total number of cFos+ cells in NTS excluding GLP-1/cFos+ cells (**g**). Mean cell volume of GLP-1-producing neurons in the studied groups (**h**). All data are presented as mean ± SD. Unpaired two-tailed *t* test with Welch’s correction was used to compare SD vs. HFD group. Ordinary one-way ANOVA followed by uncorrected Fisher’s LSD test was used to compare HFD vs. HFD-Lina vs. HFD-Gli groups. ¤, & denote *p* < 0.05, @@ denotes *p* < 0.01, *** denotes *p* < 0.001, **** denotes *p* < 0.0001, *n* = 6–7. White dotted lines outline NTS borders. Orange rectangles indicate areas with positively stained cells. *Gli* glimepiride, *GLP-1* glucagon-like peptide-1, *HFD* high-fat diet, *Lina* linagliptin, *SD* standard diet

No difference was detected in the total number of NTS GLP-1-producing neurons between HFD- and SD-fed mice, suggesting no change in GLP-1 expression or cell death. Additionally, no effect of linagliptin or glimepiride was recorded (Fig. 2a, b). However, we observed an increased number of NTS cFos+ cells in the HFD compared to SD group (*p*=0.0008), suggesting an increased cellular activation in NTS, with no additional effects of the anti-diabetic drugs (Fig. 2c, d). The number of double-stained GLP-1/cFos+ cells was increased in the HFD group (*p* < 0.0001 compared to the SD group) (Fig. 2e, f) indicating increased activation of GLP-1+ cells. Both linagliptin (*p*=0.0426) and glimepiride (*p*=0.0158), partially, but significantly, counteracted this effect (Fig. 2f). To examine if these effects occurred specifically in GLP-1-producing neurons, we subtracted the fraction of cFos/GLP-1+ neurons from the total number of NTS cFos+/activated cells. The effects of HFD, linagliptin, and glimepiride were lost after this subtraction (Fig. 2g), indicating that the recorded increase/decrease in the number of NTS cFos+ cells (and, thus, indirectly in their activation) induced by HFD and anti-hyperglycemic treatments, respectively, occurs specifically in GLP-1-producing neurons.

Twelve months of HFD feeding had no effect on the mean volume of activated GLP-1/cFos+ cells (Fig. 2h). However, we recorded a significant decrease in this parameter in the HFD-Gli group (*p*=0.0215 compared to HFD, and *p*=0.0037 compared to HFD-Lina), suggesting increased cell atrophy.

**Fig 3:**
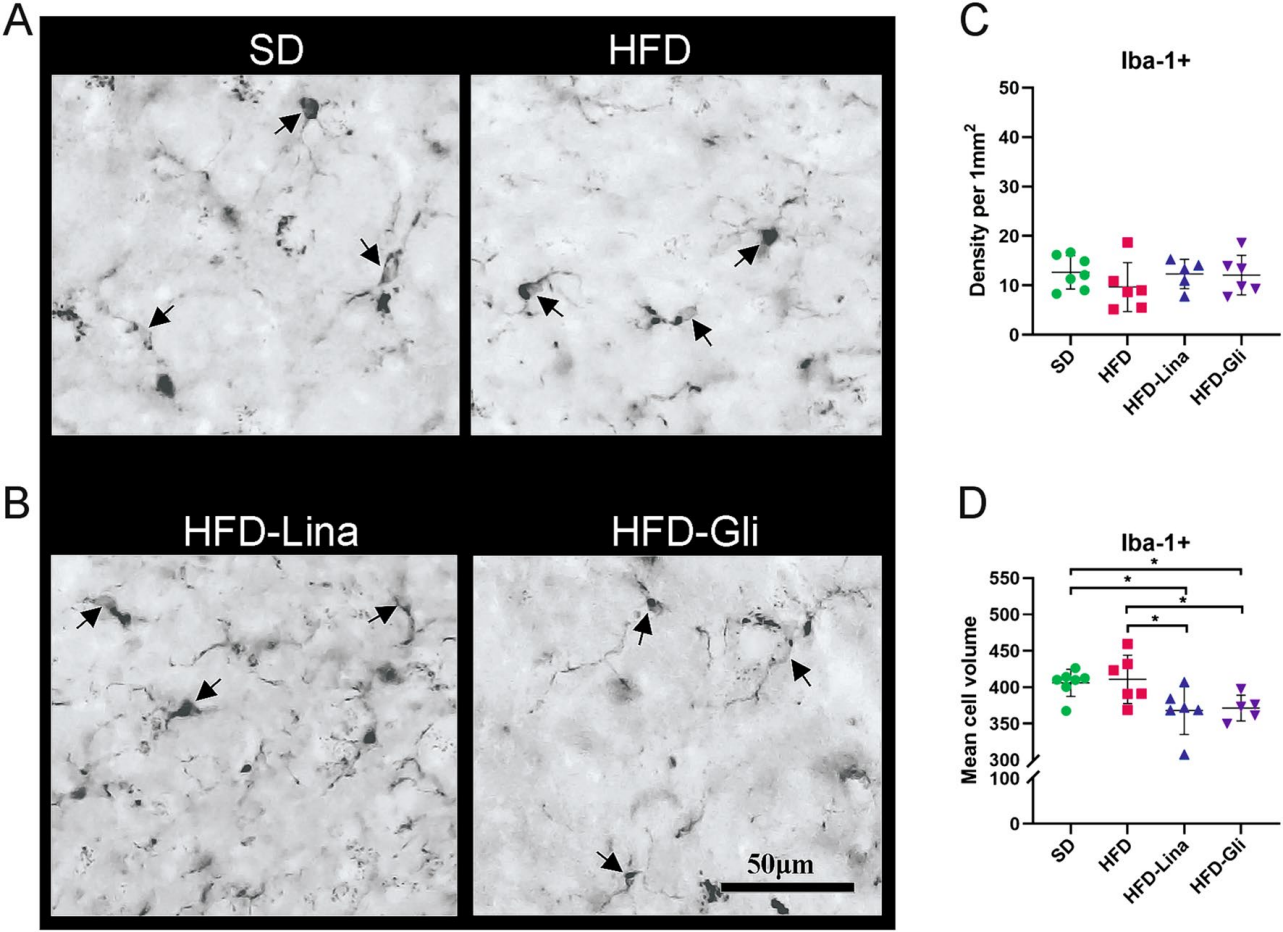
Linagliptin and glimepiride reduce neuroinflammation in NTS of middle-aged mice. Representative microphotographs (arrows indicate positive cells) (**a, b**), density (**c**), and mean volume (**d**) of Iba-1+ cells in the NTS. All data are presented as Mean ± SD. Unpaired two-tailed *t* test with Welch’s correction was used to compare SD vs. HFD group. Ordinary one-way ANOVA followed by uncorrected Fisher’s LSD test was used to compare HFD vs. HFD-Lina vs. HFD-Gli groups. ¤, & denote *p* < 0.05, *n* = 5–7. *Gli* glimepiride, *HFD* high-fat diet, *Iba-1* ionized calcium-binding adaptor molecule 1, *Lina* linagliptin, *SD* standard diet

No significant difference in Iba+ cells density in NTS between SD- and HFD-fed mice (Fig. 3a, c), or in response to anti-hyperglycemic drugs was detected (Fig. 3b, c). Interestingly, both linagliptin and glimepiride significantly reduced the volume of NTS Iba+ cells in HFD-fed mice (*p*=0.0252 and *p*=0.0449, respectively) **(**Fig. 3a-d**)**, suggesting decreased neuroinflammation. We observed no CD68+ cells in any of the studied groups (data not shown).

## Discussion

We hypothesized that obesity-induced T2D obtained after 12 months of HFD feeding impairs the function of NTS GLP-1-producing neurons by altering their basal activation (i.e., not pharmacologically stimulated), GLP-1 expression, and/or by inducing cellular atrophy and inflammation. Our results do not indicate significant changes in the number of GLP-1-expressing cells or atrophy/cellular death in response to HFD. However, we detected decreased volume of NTS GLP-1-producing neurons induced by glimepiride, possibly revealing partial atrophy of these cells and perhaps impaired GLP-1 production after sulfonylurea treatment. These effects may result from sulfonylurea-induced inhibition of neuronal ATP-sensitive potassium (K_ATP_) channels (Rosati et al. 1998) leading eventually to apoptosis (Zhang et al. 2018), whose cell shrinkage is one of the most characteristic features (Saraste and Pulkki 2000). However, decrease in cell volume can also occur independently from apoptosis (Bortner and Cidlowski 2003). Future studies are needed to investigate the mechanisms.

Interestingly, in obese/T2D mice we recorded increased cFos expression in GLP-1-producing neurons indicating an increased activation/depolarization. Although unlikely, it cannot be excluded that the increased activation of GLP-1-producing neurons is not the result of elevated food consumption in HFD-fed mice (Merchenthaler et al. 1999). Whether this finding represents a pathophysiological process, similar to β-cell hypersecretion prior to β-cell failure in T2D (Aston-Mourney et al. 2008), remains to be investigated by performing studies assessing cFos under direct pharmacological challenge. The fact that both linagliptin and glimepiride decreased obesity/T2D-induced c-Fos expression in GLP-1-producing neurons may indicate that this effect occurs via glycemia regulation, since these drugs normalize glycemia through unrelated mechanisms.

No difference in neuroinflammation between SD and HFD-fed mice was detected after 12 months in NTS, which may indicate lack of an association between changes in the basal activity of GLP-1-producing neurons induced by HFD and increased neuroinflammation Though speculative, HFD may increase neuroinflammation at earlier time points, as previously reported (Speretta et al. 2019; Butler et al. 2020). This effect could be masked at 12 months by increased neuroinflammation during the normal aging process (Cribbs et al. 2012). A decreased volume of Iba+ cells was recorded in response to linagliptin and glimepiride suggesting decreased T2D-induced NTS neuroinflammation after 12 weeks of glycemia normalization. Future studies will have to demonstrate whether decreased NTS neuroinflammation of obese/T2D mice after glycemia normalization could represent a causal factor or a consequence of the normalization of the T2D-induced activation of NTS GLP-1-producing neurons.

The preliminary findings of this short communication need to be confirmed using direct methods to assess NTS neuronal activation. Additionally, electrophysiological studies should be performed to investigate whether the changes in the activation of NTS GLP-1-producing neurons are reflected in altered GLP-1R activation/GLP-1 content in areas targeted by these cells.

In conclusion, our study provides new insights into the effects of obesity-induced T2D on NTS GLP-1-producing neurons that could lead to development of new strategies to improve GLP-1-mediated metabolic control and neuronal function in the CNS.

## Supplementary Information

Below is the link to the electronic supplementary material.

Supplementary file1 (DOCX 20 KB)

## Data Availability

All original data from this article are available upon reasonable request.

## References

[CR1] Aston-Mourney K, Proietto J, Morahan G, Andrikopoulos S (2008). Too much of a good thing: why it is bad to stimulate the beta cell to secrete insulin. Diabetologia.

[CR2] Balls M (2009). FRAME, animal experimentation and the three Rs: past, present and future. Altern Lab Anim.

[CR3] Bortner CD, Cidlowski JA (2003). Uncoupling cell shrinkage from apoptosis reveals that Na+ influx is required for volume loss during programmed cell death. J Biol Chem.

[CR4] Butler MJ, Perrini AA, Eckel LA (2020). Estradiol treatment attenuates high fat diet-induced microgliosis in ovariectomized rats. Horm Behav.

[CR5] Chalichem NSS, Gonugunta C, Krishnamurthy PT, Duraiswamy B (2017). DPP4 Inhibitors can be a drug of choice for type 3 diabetes: a mini review. Am J Alzheimers Dis Other Demen.

[CR6] Christensen M, Sparre-Ulrich AH, Hartmann B, Grevstad U, Rosenkilde MM, Holst JJ, Vilsboll T, Knop FK (2015). Transfer of liraglutide from blood to cerebrospinal fluid is minimal in patients with type 2 diabetes. Int J Obes (Lond).

[CR7] Cork SC, Richards JE, Holt MK, Gribble FM, Reimann F, Trapp S (2015). Distribution and characterisation of Glucagon-like peptide-1 receptor expressing cells in the mouse brain. Mol Metab.

[CR8] Cribbs DH, Berchtold NC, Perreau V, Coleman PD, Rogers J, Tenner AJ, Cotman CW (2012). Extensive innate immune gene activation accompanies brain aging, increasing vulnerability to cognitive decline and neurodegeneration: a microarray study. J Neuroinflammation.

[CR9] Darsalia V, Klein T, Nystrom T, Patrone C (2017). Glucagon-like receptor 1 agonists and DPP-4 inhibitors: anti-diabetic drugs with anti-stroke potential. Neuropharmacology.

[CR10] Darsalia V, Johansen OE, Lietzau G, Nystrom T, Klein T, Patrone C (2019). Dipeptidyl Peptidase-4 Inhibitors for the Potential Treatment of Brain Disorders; a mini-review with special focus on linagliptin and stroke. Front Neurol.

[CR11] Deacon CF, Holst JJ (2013). Dipeptidyl peptidase-4 inhibitors for the treatment of type 2 diabetes: comparison, efficacy and safety. Expert Opin Pharmacother.

[CR12] Drucker DJ (2018). Mechanisms of action and therapeutic application of glucagon-like peptide-1. Cell Metab.

[CR13] During MJ, Cao L, Zuzga DS, Francis JS, Fitzsimons HL, Jiao X, Bland RJ, Klugmann M, Banks WA, Drucker DJ, Haile CN (2003). Glucagon-like peptide-1 receptor is involved in learning and neuroprotection. Nat Med.

[CR14] Gault VA, Holscher C (2018). GLP-1 receptor agonists show neuroprotective effects in animal models of diabetes. Peptides.

[CR15] Graham DL, Durai HH, Trammell TS, Noble BL, Mortlock DP, Galli A, Stanwood GD (2020). A novel mouse model of glucagon-like peptide-1 receptor expression: a look at the brain. J Comp Neurol.

[CR16] Gundersen HJ, Bagger P, Bendtsen TF, Evans SM, Korbo L, Marcussen N, Moller A, Nielsen K, Nyengaard JR, Pakkenberg B (1988). The new stereological tools: disector, fractionator, nucleator and point sampled intercepts and their use in pathological research and diagnosis. APMIS.

[CR17] Holt MK, Richards JE, Cook DR, Brierley DI, Williams DL, Reimann F, Gribble FM, Trapp S (2019). Preproglucagon neurons in the nucleus of the solitary tract are the main source of brain GLP-1, mediate stress-induced hypophagia, and limit unusually large intakes of food. Diabetes.

[CR18] Hunter K, Holscher C (2012). Drugs developed to treat diabetes, liraglutide and lixisenatide, cross the blood brain barrier and enhance neurogenesis. BMC Neurosci.

[CR19] Khunti K, Chatterjee S, Gerstein HC, Zoungas S, Davies MJ (2018). Do sulphonylureas still have a place in clinical practice?. Lancet Diabetes Endocrinol.

[CR20] Kieffer TJ, Francis Habener J (1999). The Glucagon-Like Peptides. Endocr Rev.

[CR21] Larsen PJ, Tang-Christensen M, Holst JJ, Orskov C (1997). Distribution of glucagon-like peptide-1 and other preproglucagon-derived peptides in the rat hypothalamus and brainstem. Neuroscience.

[CR22] Lietzau G, Magni G, Kehr J, Yoshitake T, Candeias E, Duarte AI, Pettersson H, Skogsberg J, Abbracchio MP, Klein T, Nystrom T, Ceruti S, Darsalia V, Patrone C (2020). Dipeptidyl peptidase-4 inhibitors and sulfonylureas prevent the progressive impairment of the nigrostriatal dopaminergic system induced by diabetes during aging. Neurobiol Aging.

[CR23] Llewellyn-Smith IJ, Reimann F, Gribble FM, Trapp S (2011). Preproglucagon neurons project widely to autonomic control areas in the mouse brain. Neuroscience.

[CR24] Merchenthaler I, Lane M, Shughrue P (1999). Distribution of pre-pro-glucagon and glucagon-like peptide-1 receptor messenger RNAs in the rat central nervous system. J Comp Neurol.

[CR25] Muller TD, Finan B, Bloom SR, D'Alessio D, Drucker DJ, Flatt PR, Fritsche A, Gribble F, Grill HJ, Habener JF, Holst JJ, Langhans W, Meier JJ, Nauck MA, Perez-Tilve D, Pocai A, Reimann F, Sandoval DA, Schwartz TW, Seeley RJ, Stemmer K, Tang-Christensen M, Woods SC, DiMarchi RD, Tschop MH (2019). Glucagon-like peptide 1 (GLP-1). Mol Metab.

[CR26] Rinaman L (1999). Interoceptive stress activates glucagon-like peptide-1 neurons that project to the hypothalamus. Am J Physiol.

[CR27] Rosati B, Rocchetti M, Zaza A, Wanke E (1998). Sulfonylureas blockade of neural and cardiac HERG channels. FEBS Lett.

[CR28] Saraste A, Pulkki K (2000). Morphologic and biochemical hallmarks of apoptosis. Cardiovasc Res.

[CR29] Speretta GF, Ruchaya PJ, Delbin MA, Melo MR, Li H, Menani JV, Sumners C, Colombari E, Bassi M, Colombari DSA (2019). Importance of AT1 and AT2 receptors in the nucleus of the solitary tract in cardiovascular responses induced by a high-fat diet. Hypertens Res.

[CR30] Zhang R, Zhou X, Shen X, Xie T, Xu C, Zou Z, Dong J, Liao L (2018). Different sulfonylureas induce the apoptosis of proximal tubular epithelial cell differently via closing KATP channel. Mol Med.

