# Supplementary material for: A High-Fat Diet Increases Activation of the Glucagon-Like Peptide-1-Producing Neurons in the Nucleus Tractus Solitarii: an Effect that is Partially Reversed by Drugs Normalizing Glycemia
Source: Cell Mol Neurobiol. 2021 Apr 3;42(6):1995–2002. doi: 10.1007/s10571-021-01079-2 (PMC9239971; doi:10.1007/s10571-021-01079-2)
Supplement: Supplementary file 1 — Supplementary file1 (DOCX 20 KB) [file 10571_2021_1079_MOESM1_ESM.docx]

**Supplementary Information**

**A high-fat diet increases activation of the glucagon-like peptide-1-producing neurons in the nucleus tractus solitarii; an effect that is partially reversed by drugs normalizing glycaemia**

*Grazyna Lietzau^1,2^, Stelia Ntika^3,4^, Hiranya Pintana^1^, Linda Tracy^4^, Thomas Klein^5^, Thomas Nyström^1^, Vladimer Darsalia^1^, Cesare Patrone^1^, *Camilla Krizhanovskii^3,4^

^1^Karolinska Institutet, Department of Clinical Science and Education, Sodersjukhuset, Internal Medicine, Stockholm, Sweden

^2^Medical University of Gdansk, Faculty of Medicine, Department of Anatomy and Neurobiology, Gdansk, Poland

^3^Karolinska Institutet, Department of Molecular Medicine and Surgery, Stockholm, Sweden

^4^Södertälje hospital, Department of Research, Södertälje, Sweden

^5^Boehringer Ingelheim Pharma GmbH & Co KG, Biberach, Germany

***Material collection and immunohistochemistry***

After 12 months, mice were administered with a lethal dose of sodium pentobarbital and perfused transcardially with saline and 4% paraformaldehyde (PFA). Brains were extracted and post-fixed in 4% PFA overnight at 4°C, submersed in 20% sucrose in phosphate buffer for 3 days, cut in 30-µm-thick coronal sections on sliding microtome (Leica, Germany), and stained as free-floating. For GLP-1 and cFos, antigen retrieval protocol was applied (incubation in 10mM sodium citrate buffer, pH 6.0 at temp. gradually increasing from 70°C to 95°C, when the 95°C were reached the sections were incubated for 25 min.). For immunofluorescent detection, the following primary antibodies were applied: anti- GLP-1 (1:1200; ab22625, Abcam, UK), anti-cFos (1:500; ab208942, Abcam), and anti-Iba-1 (1:600; ab5076, Abcam). Sections were incubated with the primary antibodies at 4°C in phosphate buffer containing 0.25% Triton-X, 5% normal horse serum and normal goat serum for 48 hrs. Alexa Fluor488 and Alexa Fluor594 (horse or goat-derived) secondary antibodies (1:200; Vector Laboratories) were used.

GLP-1-producing neurons were quantified on 4 coronal sections (30µm) located at a distance between -7.48mm and -8.00mm from Bregma. To evaluate potential activation of microglia in the NTS, we quantified density and measured the mean volume of Iba-1+ cells (marker for ramified and activated microglia) and the total number of CD68+ (marker for activated microglia/microphage lineage) cells. The Iba-1 images (Fig.3) have been converted to grey-scale for better cell visibility.
